# Supplementary material for: Hot spot prediction in protein-protein interactions by an ensemble system
Source: BMC Syst Biol. 2018 Dec 31;12(Suppl 9):132. doi: 10.1186/s12918-018-0665-8 (PMC6311905; doi:10.1186/s12918-018-0665-8)
Supplement: Supplementary file 2 — All relevant MATLAB code. A simple Matlab implement of our predictor. (ZIP 19,742 kb) [file 12918_2018_665_MOESM2_ESM.zip › Additional file 2/Read ASA/ASA website/dbMPIKT/4YH7B.html]

 NetSurfP - Protein Surface Accessibility and Secondary Structure Predictions

  

|  |  |  |
| --- | --- | --- |
|  |  | NetSurfP - Protein Surface Accessibility and Secondary Structure Predictions  Technical University of Denmark |

  


---

```
# For publication of results, please cite:
# A generic method for assignment of reliability scores applied to solvent accessibility predictions.
# Bent Petersen, Thomas Nordahl Petersen, Pernille Andersen, Morten Nielsen and Claus Lundegaard
# BMC Structural Biology 2009, 9:51 doi:10.1186/1472-6807-9-51
#
# Column 1: Class assignment - B for buried or E for Exposed - Threshold: 25% exposure, but not based on RSA
# Column 2: Amino acid
# Column 3: Sequence name
# Column 4: Amino acid number
# Column 5: Relative Surface Accessibility - RSA
# Column 6: Absolute Surface Accessibility
# Column 7: Z-fit score for RSA prediction
# Column 8: Probability for Alpha-Helix
# Column 9: Probability for Beta-strand
# Column 10: Probability for Coil
E A  4YH7B                  1    0.843  92.855  -2.092   0.003   0.003   0.994
E Q  4YH7B                  2    0.579 103.445  -0.098   0.018   0.088   0.893
E P  4YH7B                  3    0.551  78.187  -1.001   0.019   0.141   0.840
E A  4YH7B                  4    0.378  41.700  -0.923   0.020   0.205   0.775
E A  4YH7B                  5    0.441  48.609  -0.967   0.021   0.279   0.699
E R  4YH7B                  6    0.406  93.066   0.230   0.004   0.420   0.576
E D  4YH7B                  7    0.596  85.912   0.825   0.003   0.718   0.279
B L  4YH7B                  8    0.123  22.595   1.119   0.001   0.900   0.099
E K  4YH7B                  9    0.418  85.941   1.085   0.001   0.959   0.040
B V  4YH7B                 10    0.075  11.543  -0.112   0.001   0.959   0.040
B V  4YH7B                 11    0.237  36.396   0.852   0.001   0.959   0.040
B T  4YH7B                 12    0.137  19.057   0.305   0.018   0.846   0.136
E K  4YH7B                 13    0.416  85.633   0.004   0.021   0.451   0.528
E R  4YH7B                 14    0.371  84.959  -0.451   0.021   0.279   0.699
E G  4YH7B                 15    0.475  37.414  -0.763   0.020   0.205   0.775
E S  4YH7B                 16    0.435  50.970  -0.962   0.019   0.141   0.840
B A  4YH7B                 17    0.285  31.374  -0.787   0.019   0.141   0.840
E D  4YH7B                 18    0.670  96.605  -0.298   0.019   0.141   0.840
E G  4YH7B                 19    0.520  40.885   0.195   0.018   0.088   0.893
B C  4YH7B                 20    0.235  32.994   0.151   0.018   0.047   0.935
B T  4YH7B                 21    0.238  33.052  -0.355   0.004   0.197   0.799
E D  4YH7B                 22    0.558  80.465   0.826   0.004   0.616   0.381
B W  4YH7B                 23    0.115  27.778   0.501   0.003   0.718   0.279
E S  4YH7B                 24    0.409  47.958  -0.164   0.004   0.514   0.481
E V  4YH7B                 25    0.444  68.243   0.142   0.005   0.336   0.660
E D  4YH7B                 26    0.538  77.583   0.660   0.004   0.197   0.799
B I  4YH7B                 27    0.181  33.393  -1.076   0.005   0.045   0.951
E K  4YH7B                 28    0.625 128.583   0.779   0.004   0.197   0.799
E K  4YH7B                 29    0.605 124.469   0.177   0.004   0.514   0.481
B Y  4YH7B                 30    0.165  35.196   1.168   0.002   0.816   0.182
E Q  4YH7B                 31    0.504  90.086   1.076   0.001   0.959   0.040
B V  4YH7B                 32    0.075  11.512   0.263   0.001   0.900   0.099
E L  4YH7B                 33    0.423  77.451   1.012   0.004   0.616   0.381
E V  4YH7B                 34    0.532  81.784   0.621   0.004   0.138   0.858
E G  4YH7B                 35    0.754  59.340  -0.836   0.005   0.015   0.979
E E  4YH7B                 36    0.428  74.807   1.239   0.004   0.085   0.910
E P  4YH7B                 37    0.544  77.222   1.550   0.004   0.616   0.381
B V  4YH7B                 38    0.043   6.671   0.545   0.001   0.959   0.040
E R  4YH7B                 39    0.359  82.097   1.928   0.001   0.959   0.040
B I  4YH7B                 40    0.037   6.771   0.432   0.001   0.959   0.040
E K  4YH7B                 41    0.358  73.579   1.669   0.001   0.959   0.040
B C  4YH7B                 42    0.019   2.738   1.051   0.001   0.900   0.099
E A  4YH7B                 43    0.395  43.584   1.149   0.001   0.900   0.099
B L  4YH7B                 44    0.070  12.835  -1.096   0.003   0.718   0.279
E F  4YH7B                 45    0.412  82.588  -0.030   0.021   0.451   0.528
E Y  4YH7B                 46    0.507 108.239  -1.652   0.019   0.141   0.840
E G  4YH7B                 47    0.520  40.916  -1.553   0.052   0.084   0.864
E Y  4YH7B                 48    0.415  88.621  -1.461   0.064   0.216   0.721
E I  4YH7B                 49    0.429  79.365  -1.183   0.022   0.359   0.619
E R  4YH7B                 50    0.418  95.630  -1.075   0.022   0.359   0.619
E T  4YH7B                 51    0.376  52.082  -0.949   0.022   0.359   0.619
E N  4YH7B                 52    0.422  61.722  -1.045   0.066   0.296   0.638
B Y  4YH7B                 53    0.263  56.118  -1.049   0.064   0.216   0.721
E S  4YH7B                 54    0.437  51.216  -1.182   0.066   0.296   0.638
B L  4YH7B                 55    0.311  56.962  -1.610   0.064   0.216   0.721
E A  4YH7B                 56    0.538  59.299  -1.415   0.019   0.141   0.840
E Q  4YH7B                 57    0.527  94.104  -2.580   0.018   0.047   0.935
E S  4YH7B                 58    0.448  52.541  -0.952   0.005   0.015   0.979
E A  4YH7B                 59    0.331  36.520  -1.012   0.005   0.045   0.951
E G  4YH7B                 60    0.521  41.034   0.416   0.004   0.085   0.910
B L  4YH7B                 61    0.090  16.552   0.024   0.005   0.336   0.660
E S  4YH7B                 62    0.368  43.118   1.217   0.001   0.900   0.099
B L  4YH7B                 63    0.037   6.701   0.252   0.001   0.959   0.040
E M  4YH7B                 64    0.240  47.984   1.917   0.001   0.959   0.040
B W  4YH7B                 65    0.027   6.421   0.790   0.001   0.959   0.040
B Y  4YH7B                 66    0.222  47.420   0.904   0.001   0.959   0.040
B K  4YH7B                 67    0.179  36.820   0.744   0.002   0.816   0.182
E S  4YH7B                 68    0.525  61.589   0.026   0.018   0.088   0.893
E S  4YH7B                 69    0.683  80.048  -1.939   0.018   0.019   0.964
E G  4YH7B                 70    0.567  44.631  -0.434   0.004   0.514   0.481
E P  4YH7B                 71    0.593  84.189  -0.256   0.022   0.552   0.426
B G  4YH7B                 72    0.149  11.750  -0.366   0.021   0.279   0.699
E D  4YH7B                 73    0.492  70.868  -0.222   0.020   0.205   0.775
E F  4YH7B                 74    0.539 108.197  -1.043   0.020   0.205   0.775
E E  4YH7B                 75    0.605 105.676  -1.270   0.020   0.205   0.775
E E  4YH7B                 76    0.483  84.310  -0.879   0.022   0.359   0.619
E P  4YH7B                 77    0.451  63.969  -0.602   0.022   0.552   0.426
E I  4YH7B                 78    0.333  61.605  -1.136   0.022   0.552   0.426
E A  4YH7B                 79    0.431  47.474  -1.196   0.022   0.552   0.426
E F  4YH7B                 80    0.376  75.403  -1.377   0.022   0.359   0.619
E D  4YH7B                 81    0.610  87.829  -1.954   0.004   0.138   0.858
E G  4YH7B                 82    0.489  38.461  -1.374   0.004   0.138   0.858
B S  4YH7B                 83    0.295  34.527  -1.351   0.004   0.514   0.481
E R  4YH7B                 84    0.328  75.135  -0.338   0.002   0.816   0.182
B M  4YH7B                 85    0.149  29.715  -0.635   0.001   0.959   0.040
E S  4YH7B                 86    0.320  37.551  -0.155   0.001   0.900   0.099
E K  4YH7B                 87    0.324  66.667  -0.587   0.003   0.718   0.279
E E  4YH7B                 88    0.549  95.928  -1.461   0.005   0.262   0.733
E E  4YH7B                 89    0.618 108.052  -0.551   0.005   0.045   0.951
B D  4YH7B                 90    0.166  23.949  -0.434   0.005   0.262   0.733
B S  4YH7B                 91    0.165  19.303   0.618   0.001   0.900   0.099
B I  4YH7B                 92    0.017   3.145   1.193   0.001   0.959   0.040
B W  4YH7B                 93    0.221  53.126   1.996   0.006   0.962   0.032
B F  4YH7B                 94    0.022   4.415   0.810   0.001   0.959   0.040
E R  4YH7B                 95    0.400  91.623   1.499   0.002   0.816   0.182
E P  4YH7B                 96    0.559  79.251   0.991   0.004   0.616   0.381
B T  4YH7B                 97    0.086  11.900   0.629   0.005   0.336   0.660
E L  4YH7B                 98    0.476  87.101   1.115   0.004   0.420   0.576
E L  4YH7B                 99    0.539  98.673   0.300   0.004   0.420   0.576
E Q  4YH7B                100    0.628 112.232  -0.244   0.004   0.138   0.858
B D  4YH7B                101    0.266  38.374  -0.988   0.005   0.045   0.951
E S  4YH7B                102    0.573  67.156  -0.135   0.004   0.085   0.910
B G  4YH7B                103    0.219  17.204  -1.285   0.004   0.616   0.381
E L  4YH7B                104    0.398  72.910   2.004   0.001   0.900   0.099
B Y  4YH7B                105    0.048  10.364   0.611   0.001   0.959   0.040
B A  4YH7B                106    0.257  28.321   2.022   0.001   0.959   0.040
B C  4YH7B                107    0.015   2.050   1.486   0.001   0.959   0.040
B V  4YH7B                108    0.232  35.705   1.917   0.001   0.959   0.040
B I  4YH7B                109    0.037   6.826  -0.695   0.001   0.959   0.040
E R  4YH7B                110    0.396  90.753   0.438   0.001   0.959   0.040
B N  4YH7B                111    0.144  21.111  -0.307   0.004   0.616   0.381
E S  4YH7B                112    0.602  70.601  -0.766   0.004   0.085   0.910
E T  4YH7B                113    0.520  72.096  -0.890   0.004   0.197   0.799
E Y  4YH7B                114    0.424  90.566  -1.056   0.004   0.616   0.381
E C  4YH7B                115    0.489  68.670  -0.414   0.002   0.816   0.182
E M  4YH7B                116    0.357  71.456  -0.494   0.001   0.900   0.099
E K  4YH7B                117    0.478  98.345   0.195   0.001   0.900   0.099
B V  4YH7B                118    0.147  22.517   0.510   0.001   0.959   0.040
E S  4YH7B                119    0.428  50.126   0.911   0.001   0.959   0.040
B I  4YH7B                120    0.043   7.881   0.163   0.001   0.959   0.040
E S  4YH7B                121    0.290  33.929   1.629   0.001   0.959   0.040
B L  4YH7B                122    0.021   3.863   1.086   0.001   0.959   0.040
E T  4YH7B                123    0.284  39.432   1.919   0.001   0.959   0.040
B V  4YH7B                124    0.029   4.519   0.368   0.001   0.959   0.040
E G  4YH7B                125    0.322  25.334   1.154   0.001   0.900   0.099
E E  4YH7B                126    0.327  57.214   0.496   0.004   0.616   0.381
E N  4YH7B                127    0.425  62.264   0.488   0.019   0.141   0.840
E D  4YH7B                128    0.359  51.732  -0.710   0.018   0.047   0.935
E T  4YH7B                129    0.650  90.210  -0.774   0.019   0.141   0.840
E G  4YH7B                130    0.346  27.214  -1.021   0.020   0.205   0.775
E L  4YH7B                131    0.323  59.068  -0.807   0.019   0.141   0.840
E C  4YH7B                132    0.492  69.035  -1.530   0.020   0.205   0.775
E Y  4YH7B                133    0.453  96.763  -0.222   0.019   0.141   0.840
B N  4YH7B                134    0.282  41.343  -1.801   0.018   0.088   0.893
E S  4YH7B                135    0.599  70.168  -1.434   0.018   0.088   0.893
E K  4YH7B                136    0.631 129.797  -0.525   0.053   0.043   0.903
E M  4YH7B                137    0.263  52.706  -0.435   0.018   0.047   0.935
E K  4YH7B                138    0.497 102.212  -1.297   0.018   0.088   0.893
E Y  4YH7B                139    0.432  92.340  -0.892   0.021   0.279   0.699
E F  4YH7B                140    0.467  93.727  -0.535   0.022   0.552   0.426
E E  4YH7B                141    0.243  42.505   0.307   0.018   0.846   0.136
E K  4YH7B                142    0.481  99.003   0.592   0.011   0.918   0.071
B A  4YH7B                143    0.083   9.180   0.390   0.018   0.846   0.136
E E  4YH7B                144    0.507  88.538   0.620   0.004   0.514   0.481
E L  4YH7B                145    0.442  80.930  -0.179   0.005   0.262   0.733
E S  4YH7B                146    0.622  72.852  -1.408   0.005   0.045   0.951
E K  4YH7B                147    0.509 104.742   0.152   0.004   0.138   0.858
E S  4YH7B                148    0.543  63.581   0.427   0.004   0.616   0.381
B K  4YH7B                149    0.063  12.918  -0.392   0.001   0.900   0.099
E E  4YH7B                150    0.403  70.422   1.522   0.001   0.959   0.040
B I  4YH7B                151    0.042   7.789  -0.203   0.001   0.959   0.040
E S  4YH7B                152    0.417  48.896   1.262   0.001   0.959   0.040
B C  4YH7B                153    0.031   4.409  -0.307   0.002   0.816   0.182
E R  4YH7B                154    0.425  97.440   0.493   0.004   0.616   0.381
E D  4YH7B                155    0.339  48.792  -1.127   0.004   0.514   0.481
B I  4YH7B                156    0.284  52.503  -0.124   0.021   0.451   0.528
E E  4YH7B                157    0.581 101.466  -0.767   0.021   0.279   0.699
E D  4YH7B                158    0.322  46.415  -0.666   0.021   0.451   0.528
E F  4YH7B                159    0.468  93.948  -0.224   0.023   0.655   0.322
B L  4YH7B                160    0.140  25.561  -1.608   0.022   0.552   0.426
E L  4YH7B                161    0.550 100.705  -0.495   0.022   0.359   0.619
E P  4YH7B                162    0.398  56.434  -1.937   0.004   0.085   0.910
E T  4YH7B                163    0.432  59.863  -0.304   0.005   0.045   0.951
B R  4YH7B                164    0.231  52.945  -0.294   0.005   0.045   0.951
E E  4YH7B                165    0.527  92.014   1.007   0.004   0.085   0.910
B P  4YH7B                166    0.117  16.631  -0.217   0.005   0.336   0.660
E E  4YH7B                167    0.440  76.798   1.106   0.002   0.816   0.182
B I  4YH7B                168    0.046   8.455   0.582   0.001   0.959   0.040
E L  4YH7B                169    0.331  60.533   2.095   0.001   0.959   0.040
B W  4YH7B                170    0.039   9.452   0.195   0.001   0.959   0.040
E Y  4YH7B                171    0.263  56.203   1.139   0.001   0.900   0.099
B K  4YH7B                172    0.241  49.574   0.523   0.003   0.718   0.279
E E  4YH7B                173    0.579 101.239  -0.352   0.018   0.047   0.935
E C  4YH7B                174    0.635  89.098  -1.720   0.018   0.047   0.935
E R  4YH7B                175    0.562 128.698   0.460   0.004   0.616   0.381
E T  4YH7B                176    0.504  69.933   0.633   0.004   0.616   0.381
B K  4YH7B                177    0.094  19.254   0.067   0.021   0.279   0.699
E A  4YH7B                178    0.551  60.687   0.887   0.004   0.138   0.858
E W  4YH7B                179    0.642 154.497  -0.737   0.004   0.085   0.910
E R  4YH7B                180    0.700 160.323  -0.555   0.005   0.045   0.951
E P  4YH7B                181    0.432  61.258  -1.564   0.004   0.138   0.858
E S  4YH7B                182    0.482  56.514  -0.097   0.003   0.718   0.279
B I  4YH7B                183    0.269  49.709   0.031   0.001   0.900   0.099
E V  4YH7B                184    0.388  59.682   0.277   0.001   0.900   0.099
E F  4YH7B                185    0.334  66.974  -1.117   0.003   0.718   0.279
E K  4YH7B                186    0.608 124.983  -1.374   0.004   0.197   0.799
E R  4YH7B                187    0.647 148.255  -0.520   0.005   0.015   0.979
B D  4YH7B                188    0.218  31.443  -0.299   0.004   0.138   0.858
E T  4YH7B                189    0.297  41.249   1.065   0.002   0.816   0.182
B L  4YH7B                190    0.023   4.230   0.696   0.001   0.959   0.040
E L  4YH7B                191    0.258  47.313   1.850   0.001   0.959   0.040
B I  4YH7B                192    0.036   6.586   0.382   0.001   0.959   0.040
E K  4YH7B                193    0.440  90.529   1.775   0.002   0.816   0.182
E E  4YH7B                194    0.549  95.840   0.996   0.004   0.616   0.381
B V  4YH7B                195    0.085  13.034   0.459   0.005   0.336   0.660
E K  4YH7B                196    0.449  92.421   1.117   0.005   0.336   0.660
E E  4YH7B                197    0.577 100.837   0.451   0.005   0.262   0.733
E D  4YH7B                198    0.654  94.241   0.028   0.004   0.085   0.910
B D  4YH7B                199    0.191  27.465  -1.314   0.005   0.045   0.951
E I  4YH7B                200    0.487  90.132  -0.168   0.004   0.138   0.858
B G  4YH7B                201    0.120   9.444  -2.179   0.003   0.718   0.279
E N  4YH7B                202    0.410  60.083   1.783   0.001   0.900   0.099
B Y  4YH7B                203    0.052  11.219   0.825   0.001   0.959   0.040
B T  4YH7B                204    0.251  34.772   1.847   0.001   0.959   0.040
B C  4YH7B                205    0.017   2.331   1.523   0.001   0.959   0.040
E E  4YH7B                206    0.244  42.609   2.182   0.001   0.959   0.040
B L  4YH7B                207    0.042   7.617   0.084   0.001   0.959   0.040
E K  4YH7B                208    0.351  72.118   0.481   0.001   0.959   0.040
B Y  4YH7B                209    0.171  36.585  -0.239   0.004   0.616   0.381
E G  4YH7B                210    0.633  49.849  -0.627   0.004   0.085   0.910
E G  4YH7B                211    0.439  34.518  -0.717   0.004   0.197   0.799
E F  4YH7B                212    0.398  79.818  -1.389   0.004   0.514   0.481
E V  4YH7B                213    0.488  75.036  -0.340   0.002   0.816   0.182
B V  4YH7B                214    0.159  24.484  -1.019   0.001   0.900   0.099
E R  4YH7B                215    0.445 101.951  -0.019   0.001   0.900   0.099
B R  4YH7B                216    0.202  46.189   0.308   0.001   0.959   0.040
E T  4YH7B                217    0.398  55.133   0.534   0.001   0.959   0.040
B T  4YH7B                218    0.039   5.409  -0.235   0.001   0.959   0.040
E E  4YH7B                219    0.329  57.511   1.529   0.001   0.959   0.040
B L  4YH7B                220    0.023   4.138   1.088   0.001   0.959   0.040
E T  4YH7B                221    0.274  37.976   2.125   0.001   0.959   0.040
B V  4YH7B                222    0.029   4.457   0.535   0.001   0.959   0.040
E T  4YH7B                223    0.334  46.326   1.226   0.001   0.900   0.099
B A  4YH7B                224    0.209  23.021  -0.045   0.002   0.816   0.182
E P  4YH7B                225    0.463  65.629   0.226   0.004   0.420   0.576
E L  4YH7B                226    0.467  85.434  -1.050   0.019   0.141   0.840
E T  4YH7B                227    0.506  70.238  -0.921   0.004   0.197   0.799
E D  4YH7B                228    0.557  80.220  -1.278   0.005   0.262   0.733
E K  4YH7B                229    0.433  89.150  -0.204   0.004   0.197   0.799
E P  4YH7B                230    0.313  44.471  -0.137   0.018   0.088   0.893
B P  4YH7B                231    0.105  14.829  -1.691   0.020   0.205   0.775
E K  4YH7B                232    0.472  97.111   0.655   0.021   0.756   0.223
B L  4YH7B                233    0.113  20.654  -1.050   0.018   0.846   0.136
E L  4YH7B                234    0.284  51.945  -0.464   0.021   0.756   0.223
E Y  4YH7B                235    0.362  77.274  -0.545   0.022   0.552   0.426
E P  4YH7B                236    0.341  48.331  -0.211   0.020   0.205   0.775
E M  4YH7B                237    0.406  81.181  -0.993   0.018   0.088   0.893
E E  4YH7B                238    0.641 112.053  -0.673   0.018   0.088   0.893
E S  4YH7B                239    0.643  75.360  -1.168   0.019   0.141   0.840
E K  4YH7B                240    0.497 102.315  -0.665   0.021   0.279   0.699
E L  4YH7B                241    0.350  64.122  -1.464   0.004   0.514   0.481
E T  4YH7B                242    0.430  59.683  -0.095   0.003   0.718   0.279
E V  4YH7B                243    0.388  59.697  -0.492   0.002   0.816   0.182
B Q  4YH7B                244    0.222  39.578   0.635   0.001   0.900   0.099
E E  4YH7B                245    0.429  75.034  -0.075   0.001   0.959   0.040
B T  4YH7B                246    0.095  13.176   0.220   0.001   0.900   0.099
E Q  4YH7B                247    0.466  83.210   0.420   0.003   0.718   0.279
E L  4YH7B                248    0.446  81.589   0.265   0.005   0.262   0.733
E G  4YH7B                249    0.641  50.439  -1.285   0.005   0.045   0.951
E G  4YH7B                250    0.434  34.187   0.502   0.004   0.085   0.910
E S  4YH7B                251    0.562  65.843   1.483   0.003   0.718   0.279
B A  4YH7B                252    0.038   4.210  -0.252   0.001   0.959   0.040
E N  4YH7B                253    0.367  53.670   1.883   0.001   0.959   0.040
B L  4YH7B                254    0.037   6.738   0.404   0.001   0.959   0.040
E T  4YH7B                255    0.344  47.754   1.773   0.001   0.900   0.099
B C  4YH7B                256    0.019   2.682   1.068   0.001   0.900   0.099
E R  4YH7B                257    0.384  87.844   1.789   0.001   0.900   0.099
B A  4YH7B                258    0.055   6.072  -0.542   0.001   0.959   0.040
E F  4YH7B                259    0.392  78.654   0.296   0.001   0.900   0.099
E F  4YH7B                260    0.312  62.558  -0.676   0.004   0.616   0.381
E G  4YH7B                261    0.402  31.598  -0.600   0.004   0.197   0.799
E Y  4YH7B                262    0.517 110.397  -1.541   0.004   0.085   0.910
E S  4YH7B                263    0.709  83.060  -0.949   0.005   0.045   0.951
E G  4YH7B                264    0.324  25.483  -2.102   0.005   0.015   0.979
E D  4YH7B                265    0.527  75.970  -0.213   0.005   0.015   0.979
B V  4YH7B                266    0.164  25.145  -1.013   0.004   0.085   0.910
E S  4YH7B                267    0.450  52.787   0.684   0.004   0.138   0.858
B P  4YH7B                268    0.056   7.946  -0.460   0.004   0.420   0.576
E L  4YH7B                269    0.353  64.634   1.234   0.001   0.900   0.099
B I  4YH7B                270    0.032   5.846   0.255   0.001   0.959   0.040
B Y  4YH7B                271    0.222  47.484   2.059   0.001   0.959   0.040
B W  4YH7B                272    0.025   5.916   0.917   0.001   0.959   0.040
E M  4YH7B                273    0.234  46.823   1.318   0.001   0.959   0.040
B K  4YH7B                274    0.197  40.482   1.050   0.002   0.816   0.182
E G  4YH7B                275    0.572  44.993  -0.080   0.018   0.047   0.935
E E  4YH7B                276    0.719 125.662  -1.360   0.018   0.019   0.964
E K  4YH7B                277    0.609 125.210   0.391   0.003   0.718   0.279
E F  4YH7B                278    0.494  99.045   0.483   0.002   0.816   0.182
B I  4YH7B                279    0.096  17.668  -0.069   0.022   0.552   0.426
E E  4YH7B                280    0.499  87.263  -0.061   0.022   0.359   0.619
E D  4YH7B                281    0.551  79.428  -1.449   0.019   0.141   0.840
E L  4YH7B                282    0.433  79.246  -2.119   0.018   0.047   0.935
E D  4YH7B                283    0.561  80.869  -2.146   0.018   0.047   0.935
E E  4YH7B                284    0.573 100.051  -0.986   0.020   0.205   0.775
B N  4YH7B                285    0.281  41.109  -1.178   0.004   0.514   0.481
E R  4YH7B                286    0.412  94.325  -0.666   0.003   0.718   0.279
B V  4YH7B                287    0.223  34.229  -1.096   0.018   0.846   0.136
E W  4YH7B                288    0.361  86.772  -0.745   0.003   0.718   0.279
E E  4YH7B                289    0.370  64.639  -1.404   0.004   0.514   0.481
E S  4YH7B                290    0.590  69.125  -2.252   0.004   0.197   0.799
E D  4YH7B                291    0.578  83.290  -1.480   0.005   0.262   0.733
E I  4YH7B                292    0.384  71.003  -1.351   0.004   0.616   0.381
E R  4YH7B                293    0.405  92.814  -0.835   0.002   0.816   0.182
E I  4YH7B                294    0.343  63.492  -1.001   0.011   0.918   0.071
E L  4YH7B                295    0.284  52.037  -0.672   0.011   0.918   0.071
E K  4YH7B                296    0.383  78.865  -1.163   0.002   0.816   0.182
E E  4YH7B                297    0.372  64.901  -0.932   0.004   0.616   0.381
E H  4YH7B                298    0.484  88.021  -1.559   0.004   0.514   0.481
E L  4YH7B                299    0.495  90.726  -1.627   0.005   0.336   0.660
E G  4YH7B                300    0.559  43.978  -1.726   0.004   0.197   0.799
E E  4YH7B                301    0.568  99.212  -1.265   0.005   0.336   0.660
E Q  4YH7B                302    0.469  83.853  -1.087   0.004   0.514   0.481
E E  4YH7B                303    0.377  65.809  -0.666   0.004   0.616   0.381
B V  4YH7B                304    0.250  38.410  -1.033   0.003   0.718   0.279
E S  4YH7B                305    0.337  39.508  -0.341   0.002   0.816   0.182
B I  4YH7B                306    0.062  11.489  -0.665   0.002   0.816   0.182
B S  4YH7B                307    0.158  18.471   0.523   0.001   0.900   0.099
B L  4YH7B                308    0.019   3.497   1.212   0.001   0.959   0.040
B I  4YH7B                309    0.200  36.982   1.648   0.001   0.959   0.040
B V  4YH7B                310    0.023   3.535   0.769   0.001   0.959   0.040
E D  4YH7B                311    0.416  59.888   1.475   0.002   0.816   0.182
E S  4YH7B                312    0.511  59.877   0.878   0.004   0.514   0.481
B V  4YH7B                313    0.079  12.204   0.200   0.004   0.197   0.799
E E  4YH7B                314    0.449  78.370   0.979   0.005   0.262   0.733
E E  4YH7B                315    0.566  98.950   0.489   0.005   0.262   0.733
E G  4YH7B                316    0.651  51.234   0.278   0.004   0.085   0.910
B D  4YH7B                317    0.208  29.930  -1.110   0.004   0.085   0.910
E L  4YH7B                318    0.451  82.541  -0.681   0.004   0.197   0.799
B G  4YH7B                319    0.195  15.362  -1.651   0.003   0.718   0.279
E N  4YH7B                320    0.423  61.869   1.602   0.001   0.900   0.099
B Y  4YH7B                321    0.045   9.659   0.764   0.001   0.959   0.040
E S  4YH7B                322    0.297  34.855   1.988   0.000   0.983   0.017
B C  4YH7B                323    0.018   2.485   1.376   0.000   0.983   0.017
E Y  4YH7B                324    0.265  56.566   1.922   0.001   0.959   0.040
B V  4YH7B                325    0.043   6.532  -0.496   0.001   0.959   0.040
E E  4YH7B                326    0.402  70.212   0.664   0.001   0.959   0.040
B N  4YH7B                327    0.116  17.041  -0.140   0.003   0.718   0.279
E G  4YH7B                328    0.603  47.464   0.041   0.004   0.197   0.799
E N  4YH7B                329    0.415  60.800  -0.597   0.005   0.336   0.660
E G  4YH7B                330    0.357  28.072  -1.385   0.003   0.718   0.279
E R  4YH7B                331    0.478 109.439  -0.031   0.001   0.900   0.099
B R  4YH7B                332    0.241  55.258  -0.742   0.001   0.959   0.040
E H  4YH7B                333    0.443  80.582   0.135   0.001   0.959   0.040
B A  4YH7B                334    0.161  17.742   0.587   0.001   0.959   0.040
E S  4YH7B                335    0.358  41.922   0.655   0.001   0.959   0.040
B V  4YH7B                336    0.028   4.242   0.088   0.001   0.959   0.040
E L  4YH7B                337    0.294  53.850   1.999   0.001   0.959   0.040
B L  4YH7B                338    0.022   3.955   0.817   0.001   0.959   0.040
E H  4YH7B                339    0.318  57.917   2.189   0.001   0.959   0.040
B K  4YH7B                340    0.078  16.086   0.067   0.001   0.900   0.099
E R  4YH7B                341    0.510 116.721   1.313   0.003   0.718   0.279
E K  4YH7B                342    0.486  99.908   0.175   0.005   0.262   0.733
E H  4YH7B                343    0.383  69.759  -0.128   0.005   0.015   0.979
E H  4YH7B                344    0.328  59.627  -0.402   0.005   0.015   0.979
E H  4YH7B                345    0.739 134.370   0.974   0.018   0.047   0.935
E H  4YH7B                346    0.573 104.301   0.135   0.018   0.047   0.935
E H  4YH7B                347    0.555 101.009  -1.465   0.018   0.047   0.935
E H  4YH7B                348    0.668 121.491  -1.431   0.003   0.003   0.994

Explain the output.  Go back.
```

---
